# Predictive Factors of Mental Health in Athletes from the Paralympic Preparation Program During Social Isolation: The Role of Sleep, Competitive Status, and Motivation

**DOI:** 10.3390/sports14040160

**Published:** 2026-04-17

**Authors:** Eduarda Coelho, Carla Lourenço, Antonino Pereira, Maria Isabel Mourão-Carvalhal, Sandra Fonseca

**Affiliations:** 1 Research Center in Sports Sciences, Health Sciences and Human Development (CIDESD), 5001-801 Vila Real, Portugal; ecoelho@utad.pt (E.C.); mimc@utad.pt (M.I.M.-C.); 2 University of Beira Interior (UBI), 6201-001 Covilhã, Portugal; carla.cvlourenco@gmail.com; 3 Centre for Studies in Education and Innovation (CI&DEI), School of Education, Polytechnic Institute of Viseu, 3504-510 Viseu, Portugal; apereira@esev.ipv.pt; 4 Department of Sports, Exercise and Health, University of Trás-os-Montes e Alto Douro, 5000-801 Vila Real, Portugal

**Keywords:** paralympic sport, mental health, linear regression, COVID-19, sleep, group effect

## Abstract

Background: The COVID-19 pandemic posed unprecedented challenges for elite athletes, disrupting training routines and affecting mental health. This study examined the impact of social isolation on training, sleep, motivation, and psychological well-being among Portuguese Paralympic athletes. Methods: Forty-five athletes (31.36 ± 10.81 years) from the Paralympic Preparation Program participated, divided into the Paralympic Preparation Project (PPP; *n* = 21) and the Paralympic Hopes and Talents Project (PHTP; *n* = 24). Training routines before and during social isolation were compared. Sleep duration, training motivation, and mental health were assessed using the Mental Health Inventory-5 (MHI-5). The analysis employed paired and independent *t*-tests and a multiple linear regression (Enter method) to examine predictors of mental health. Results: Daily training duration declined by 34.3% (*p* < 0.001), though weekly frequency remained stable (*p* = 0.061). PPP athletes reported higher mental health scores than PHTP athletes (66.24 vs. 60.37; *p* = 0.048). The regression model explained 42.8% of the variance in mental health (R^2^ = 0.428). Sleep duration was positively associated with mental health and showed the highest standardized coefficient in the regression (β = 0.351; *p* = 0.008), followed by PPP status (β = 0.315; *p* = 0.024) and motivation (β = 0.278; *p* = 0.041). Conclusions: Maintaining biological routines, particularly sleep and motivation, supports mental health under social isolation. Higher well-being among PPP athletes underscores the need for targeted interventions for less experienced athletes. Sports organizations should prioritize sleep hygiene and psychological support to mitigate vulnerability during crises.

## 1. Introduction

The COVID-19 pandemic, declared by the World Health Organization on 11 March 2020 [1], caused unprecedented disruptions to sports systems worldwide. In Portugal, the declaration of a state of emergency resulted in the closure of training facilities and suspension of competitions, forcing athletes to adapt their routines to constrained home environments [2].

Evidence from studies on general athlete populations indicates that lockdown measures were associated with reductions in training volume, disruptions to daily routines, and increased psychological distress [3]. However, Paralympic athletes may have been particularly vulnerable due to their reliance on adapted infrastructures, specialized equipment, and multidisciplinary support systems [4,5].

Moreover, the postponement of the Tokyo 2020 Paralympic Games introduced uncertainty into structured preparation cycles, potentially exacerbating stress and affecting motivation and mental health [6,7]. Research specifically focusing on athletes with disabilities suggests increased vulnerability to anxiety, sleep disturbances, and demotivation during confinement [8,9,10,11].

Despite these challenges, sport continues to be an important protective factor for psychological well-being. Regular training is linked to improved mental health, self-esteem, and social integration [12]. However, pandemic-related disruptions may compromise these benefits [13].

Nevertheless, empirical evidence specifically addressing Paralympic athletes remains limited, particularly regarding how behavioral and contextual factors relate to mental health during periods of disruption.

Therefore, the present study aimed to analyze changes in training routines and examine factors associated with mental health among Portuguese Paralympic athletes during COVID-19-related restrictions.

Specifically, the study sought to:

1. Compare training routines (weekly frequency and daily duration) between the pre-pandemic period and the period of social isolation;
2. Compare mental health levels between athletes from the Paralympic Preparation Project (PPP; Projeto de Preparação Paralímpica) and the Paralympic Hopes and Talents Project (PHTP; Projeto Esperanças e Talentos Paralímpicos);
3. Examine associations between behavioral variables (sleep, motivation, and training routines) and mental health;
4. Test an exploratory regression model of athletes’ mental health.

## 2. Materials and Methods

### 2.1. Study Design

This study adopted a quantitative, cross-sectional, exploratory, and descriptive design aimed at examining the impact of COVID-19–related social isolation on training routines and mental health among Portuguese Paralympic athletes. Data collection took place during the period of mandatory social isolation in Portugal following the declaration of the national state of emergency due to the COVID-19 pandemic.

The study specifically focused on identifying behavioral and contextual factors associated with athletes’ psychological well-being during this period, including sleep duration, training motivation, and competitive status within the Paralympic Preparation Program.

In Portugal, Paralympic sport development is structured through the Portuguese Paralympic Preparation Program, a state-funded system coordinated by the Portuguese Paralympic Committee. This program provides financial, logistical, and technical support to athletes, coaches, and sport federations throughout each four-year Paralympic cycle, aiming to prepare athletes for participation in the Paralympic Games under high-performance conditions.

### 2.2. Instruments

Data were collected through a structured self-report questionnaire composed of four sections.

1. Sociodemographic and sport-related data

Participants reported basic demographic and sport-related characteristics, including age, sex, years of sport practice, and sport modality.

2. Training and sleep routines

Participants reported their weekly training frequency (days per week) and daily training duration (hours per day) at two different time points: under normal training conditions (pre-pandemic) and during the period of social isolation. It should be noted that pre-pandemic training data were collected retrospectively during the isolation period, which may introduce recall bias. Sleep behavior was assessed by asking participants to report the average number of hours slept per day during the isolation period.

3. Perceived motivation

Training motivation during the isolation period was assessed using a single self-report item: “On a scale from 1 to 10, what is your level of motivation to train at home during the isolation period?” A score of 1 indicated “very low motivation,” and a score of 10 indicated “very high motivation.” Single-item measures have been widely used in sport and exercise research as pragmatic indicators of perceived motivational states, particularly in applied contexts [14,15]. Nevertheless, given the multidimensional nature of motivation, this approach may limit the construct validity of the assessment and should be interpreted cautiously. This limitation is acknowledged in Section 4.

4. Mental health

Mental health was assessed using the Mental Health Inventory-5 (MHI-5), a widely used screening instrument for psychological well-being. The scale comprises five items assessing feelings of nervousness, calmness, sadness, happiness, and discouragement. Responses are rated on a six-point Likert scale and converted to a standardized score ranging from 0 to 100, with higher scores indicating better mental health. The MHI-5 has demonstrated good reliability and construct validity in both general and athletic populations [16] and has been validated for the Portuguese population [17].

### 2.3. Procedures

Data were collected via the Google Forms online platform. Athletes were contacted directly through the Portuguese Paralympic Committee and informed about the study objectives, the voluntary nature of participation, and the confidentiality and anonymity of their responses. Data collection was carried out during May 2020.

Participants provided informed consent prior to completing the questionnaire. The study was conducted in accordance with the ethical principles of the Declaration of Helsinki and was approved by the Ethics Committee of the University of Trás-os-Montes and Alto Douro.

### 2.4. Statistical Analysis

All statistical analyses were performed using IBM SPSS Statistics (version 28.0). The level of statistical significance was set at *p* < 0.05.

Descriptive statistics were calculated for all variables, including means and standard deviations. Data normality was assessed using the Shapiro–Wilk test.

Comparisons between pre-pandemic and isolation training routines were performed using paired-samples Student’s *t*-tests. Differences between athletes from the PPP and the PHTP were examined using independent-samples *t*-tests, with homogeneity of variances assessed through Levene’s test.

For all mean comparisons, 95% confidence intervals (95% CI) were calculated, and effect sizes were estimated using Cohen’s d, interpreted as small (0.20), medium (0.50), and large (0.80).

To explore relationships between variables, Pearson’s correlation coefficients (r) were computed. Only variables showing significant associations with mental health (MHI-5) were considered for inclusion in the exploratory regression model.

A multiple linear regression analysis was conducted to examine associations between behavioral and contextual variables and mental health. Predictor variables were selected based on both theoretical relevance and significant correlations with the outcome variable, following recommendations for exploratory regression models in behavioral research.

Model adequacy was evaluated through residual diagnostics, including assessment of normality and homoscedasticity, the Durbin–Watson statistic for independence of errors, and Tolerance and Variance Inflation Factor (VIF) values to verify the absence of multicollinearity.

Standardized β coefficients were reported, along with 95% confidence intervals for unstandardized B coefficients.

### 2.5. Participants

Participants were eligible if they were officially enrolled in the Portuguese Paralympic Preparation Program and actively engaged in training during the study period. Recruitment was conducted through the Portuguese Paralympic Committee.

The sample was obtained through convenience sampling and consisted of 45 Portuguese athletes (32 men and 13 women) with a mean age of 31.36 ± 10.81 years.

Participants were enrolled in the Portuguese Paralympic sport system and classified according to their integration within two competitive development levels of the Portuguese Paralympic Preparation Program. The PPP includes high-performance athletes preparing for participation in the Paralympic Games, whereas the PHTP focuses on the identification and development of emerging athletes with potential to reach the Paralympic level.

Accordingly, the sample comprised athletes from the PPP (*n* = 21) and the PHTP (*n* = 24).

The athletes had an average sport experience of 10.53 ± 6.85 years and represented several Paralympic sports, including athletics (22.2%), boccia (22.2%), swimming (17.7%), goalball (11.1%), cycling (8.9%), badminton (6.7%), canoeing (4.5%), triathlon (2.2%), equestrian (2.2%), and shooting (2.2%) (Table 1).

## 3. Results

Only one athlete suspended training during social isolation. Comparison between pre-isolation and isolation periods (Table 2) revealed no significant change in weekly training frequency (5.13 ± 1.12 vs. 4.84 ± 1.43; *p* = 0.061). However, daily training duration decreased significantly, with an average reduction of 34.3%, from 2.80 ± 1.12 h to 1.84 ± 0.94 h per day (*p* < 0.001).

Perceived motivation to train at home was moderate (6.13 ± 2.12 on a 1–10 scale).

Comparison between competitive groups showed that PPP athletes were older (35.81 ± 10.2 vs. 27.46 ± 9.9; *p* = 0.011), had more sporting experience (13.10 ± 7.4 vs. 8.29 ± 5.5; *p* = 0.026), and exhibited significantly higher mental health scores (MHI-5: 66.24 ± 12.9 vs. 60.37 ± 15.9; *p* = 0.048) than PHTP athletes (Table 3).

Mental health correlated positively with all behavioral variables, showing moderate associations with motivation (r = 0.451), sleep duration (r = 0.435), and competitive status (r = 0.321). Training duration during isolation was moderately associated with motivation (r = 0.488) and mental health (r = 0.382) (Table 4). Confidence intervals indicated that most correlations were small to moderate in magnitude, with several excluding zero, supporting the robustness of the observed associations.

The multiple linear regression model explained 42.8% of the variance in mental health (R^2^ = 0.428) (Table 5). Sleep duration emerged as the strongest predictor of mental health in the model (β = 0.351), followed by competitive status (β = 0.315) and motivation (β = 0.278). Training duration during isolation showed a positive trend but did not reach statistical significance (β = 0.241; *p* = 0.051).

Residual diagnostics indicated no major violations of regression assumptions. The visual inspection of residual plots suggested approximate normality and homoscedasticity. The Durbin–Watson statistic was close to 2, indicating independence of errors. Additionally, tolerance values were above 0.70 and Variance Inflation Factor (VIF) values were below 2, suggesting the absence of multicollinearity.

## 4. Discussion

The present study investigated the impact of COVID-19–related social isolation on training routines and mental health among athletes enrolled in the Portuguese Paralympic Preparation Program, while also exploring behavioral predictors of psychological well-being. The results yielded three main findings: (i) a significant reduction in daily training duration during isolation, (ii) higher mental health levels among athletes from the PPP compared with those from the PHTP, and (iii) sleep duration, competitive status, and motivation emerging as significant predictors of mental health.

A central finding was the significant reduction in daily training duration, which decreased by 34.3%, from 2.80 ± 1.12 h to 1.84 ± 0.94 h per day (*p* < 0.001). In contrast, weekly training frequency showed no statistically significant change (5.13 ± 1.12 vs. 4.84 ± 1.43 sessions per week; *p* = 0.061). This pattern suggests that although athletes attempted to maintain regular training routines during social isolation, the restrictions imposed by lockdown measures limited the duration of training sessions. Similar patterns have been reported internationally [12,18]. Pillay et al. [18] observed that many elite athletes trained less than one hour per day during lockdown, while Puce et al. [12] documented substantial reductions in weekly training volume among athletes with disabilities. For Paralympic athletes, these disruptions may be intensified by reliance on adapted environments, specialized equipment, and technical support staff, which are often difficult to replicate in home-based settings.

Despite these constraints, the maintenance of training frequency suggests a deliberate effort to preserve routine structure. Upholding consistent routines may represent an adaptive coping strategy that helps sustain athletic identity and engagement with sport [19]. Because athletes often strongly identify with their sporting role, disruptions to training and competition may threaten this identity and thereby increase psychological vulnerability. In this context, the preservation of training frequency observed in the current study may reflect an effort to maintain continuity in athletes’ professional and personal identities during a period of profound uncertainty.

The average mental health score observed in the sample (63.11 ± 14.8) indicates moderate levels of psychological well-being. Although baseline pre-pandemic data for Portuguese Paralympic athletes are unavailable, this value is seemingly lower than levels typically reported among elite athletes under stable competitive conditions. These findings align with previous research [10,20] indicating that pandemic-related uncertainty, social isolation, and the postponement of major competitions negatively impacted athletes’ psychological health. The interruption of structured preparation cycles and the loss of short-term competitive goals may undermine athletes’ perceived sense of control and future orientation, both of which are central components of psychological resilience in high-performance sport.

The analysis of competitive status revealed significant differences between PPP and PHTP athletes, with PPP athletes presenting higher mental health scores (66.24 ± 12.9 vs. 60.37 ± 15.9; *p* = 0.048). PPP athletes were also older and had more years of sport experience, suggesting that career maturity may play a protective role in coping with disruptive events. These findings support theoretical perspectives emphasizing the importance of “career resources” in elite sport contexts. Athletes competing at higher levels typically benefit from more stable financial support, access to multidisciplinary teams, and greater experience managing competitive stressors [21]. Such resources may enhance psychological resilience when facing unexpected challenges such as those posed by the COVID-19 pandemic [10].

Correlational analyses further demonstrated that mental health was positively associated with motivation (r = 0.451), sleep duration (r = 0.435), competitive status (r = 0.321), and training duration during isolation (r = 0.382). These results highlight the interconnected role of behavioral and psychological factors in shaping athletes’ well-being during periods of social disruption. In particular, the link between motivation and training duration suggests that athletes who remained psychologically engaged with training were more likely to maintain higher levels of physical activity during isolation.

The multiple linear regression model explained 42.8% of the variance in mental health (R^2^ = 0.428), indicating a meaningful explanatory contribution of the selected predictors. Within this model, sleep duration emerged as one of the main predictors of mental health (β = 0.351; *p* = 0.008), followed by competitive status (β = 0.315; *p* = 0.024) and motivation (β = 0.278; *p* = 0.041). Sleep duration presented the highest standardized coefficient in the model, although differences between predictors were relatively small, suggesting a comparable contribution across variables. Given the sample size, these results should be interpreted with caution, and the relative importance of predictors should not be overstated. Training duration during isolation showed a positive trend but did not reach statistical significance (β = 0.241; *p* = 0.051).

The prominent role of sleep reinforces the growing recognition of sleep as a critical component of athletes’ psychophysiological functioning [21,22]. Adequate sleep is essential for physiological recovery, cognitive performance, emotional regulation, and motivational processes [10]. Insufficient or disrupted sleep has been associated with increased fatigue, reduced concentration, mood disturbances, and decreased training engagement [13,19]. During periods of uncertainty and reduced external structure, maintaining regular sleep patterns may help stabilize circadian rhythms and support emotional regulation, thereby bolstering mental health [6,7,23].

Motivation also surfaced as an important factor associated with psychological well-being. From a theoretical perspective, this relationship can be interpreted through the framework of Self-Determination Theory, which highlights the importance of autonomy, competence, and relatedness as basic psychological needs for optimal functioning [24]. Pandemic-related restrictions may have undermined athletes’ perceived autonomy by limiting access to training facilities, reduced perceptions of competence due to altered training conditions, and weakened relatedness through social isolation from teammates and coaches. Athletes who maintained higher levels of motivation may therefore have been better able to satisfy these psychological needs, contributing to greater psychological resilience during the isolation period.

Although training duration during isolation correlated positively with mental health, it did not retain its statistical significance in the regression model. This finding suggests that training volume alone may not directly determine psychological well-being but may instead operate through motivational and recovery-related mechanisms. In this context, sleep and motivation appear to represent more proximal determinants of mental health than training duration itself.

From a broader perspective, our findings can be interpreted within a biopsychosocial framework of athlete health. According to this perspective, psychological well-being emerges from the interaction between biological, psychological, and social factors [25]. In the present study, sleep duration may represent a biological regulatory mechanism, motivation reflects psychological engagement with training, and competitive status may capture social and structural resources associated with high-performance sport systems. The combined influence of these variables suggests that mental health in Paralympic athletes cannot be understood solely through training behaviors but rather through the dynamic interaction between physiological recovery processes, motivational states, and contextual support structures.

Overall, the reported results support a multidimensional understanding of athlete well-being, highlighting that biological regulation, motivational dynamics, and structural support within sport systems interact to shape psychological health in athletes during periods of environmental disruption. Such insights underscore the importance of integrating recovery strategies, psychological support, and individualized athlete management within high-performance sport programs, particularly during times of crisis or significant changes in training environments.

These findings should be interpreted in light of several limitations. First, the relatively small sample size may limit statistical power and generalizability. Second, the cross-sectional design precludes causal inference. Moreover, the use of self-report measures, including retrospective assessment of pre-pandemic training and a single-item measure of motivation, may introduce bias. These factors should be addressed in future research using longitudinal designs and more comprehensive measurement tools.

These considerations underscore that this study provides an exploratory contribution rather than a definitive explanatory model.

### Practical Implications and Recommendations

- Sleep hygiene and circadian rhythm monitoring should be core components of athlete support, especially during social isolation.
- Early career athletes (PHTP) require proactive psychological support and mentorship from experienced athletes (PPP) to maintain motivation and athletic identity.
- Short-term goal setting and continuous feedback can mitigate feelings of isolation and loss of purpose.
- Contingency protocols should prioritize psychobiological stability, integrating mental health as a central component of high-performance sport management.

Implementing these strategies can enhance resilience and preserve psychological balance during periods of training disruption or crisis.

## 5. Conclusions

The present study provides novel evidence on the mental health of Paralympic athletes during COVID-19-related social isolation, a population that remains underrepresented in sport science research.

Our findings suggest that behavioral (sleep, motivation) and contextual (competitive status) factors are meaningfully associated with psychological well-being. Rather than training volume alone, psychobiological regulation and motivational engagement appear to play a central role in supporting mental health.

This study contributes to the literature by emphasizing the importance of a multidimensional approach to athlete health, integrating biological recovery, psychological processes, and structural support systems.

From a practical standpoint, these findings reinforce the need for targeted interventions focused on sleep hygiene, motivation, and psychological support, particularly for developing athletes.

Future research should adopt longitudinal designs and include more robust measurement approaches to further clarify these relationships.

## Figures and Tables

**Table 1:** Sample Characterization.

	N	%
Age (years)		
≤20	11	24.4
21–30	11	24.4
31–40	14	31.1
41–50	7	15.6
≥51	2	4.4
Sex		
Female	13	28.9
Male	32	71.1
Sport Modality		
Athletics	10	22.2
Boccia	10	22.2
Swimming	8	17.7
Goalball	5	11.1
Cycling	4	8.9
Badminton	3	6.7
Canoeing	2	4.5
Triathlon	1	2.2
Equestrian	1	2.2
Shooting	1	2.2
Years of Practice		
≤10	27	60
>10	18	40

Note. N = number of participants; % = relative percentage in each category.

**Table 2:** Comparison of Training Routines: Pre-Isolation vs. During Isolation.

Variable	Pre-Isolation (M ± SD)	During Isolation (M ± SD)	Difference (%)	95% CI of Difference	*p*	Cohen’s *d*
Frequency (days/week)	5.13 ± 1.12	4.84 ± 1.43	−5.6	[−0.59, 0.01]	0.061	0.23
Duration (hours/day)	2.80 ± 1.12	1.84 ± 0.94	−34.3	[0.61, 1.31]	<0.001	0.73

Note. M = mean; SD = standard deviation; 95% CI = 95% confidence interval of the mean difference; *d* = effect size (Cohen’s d). *p*-values correspond to paired-samples *t*-tests for each variable.

**Table 3:** Demographic and Sport Characteristics by Competitive Status.

Variables	Total (*n* = 45) (M ± SD)	PPP (*n* = 21) (M ± SD)	PHTP (*n* = 24) (M ± SD)	95% CI of Difference	*p*	Cohen’s *d*
Age (Years)	31.36 ± 10.8	35.81 ± 10.2	27.46 ± 9.9	[1.98, 14.72]	0.011	0.83
Years of Practice	10.53 ± 6.8	13.10 ± 7.4	8.29 ± 5.5	[0.61, 9.01]	0.026	0.74
Mental Health (MHI-5)	63.11 ± 14.8	66.24 ± 12.9	60.37 ± 15.9	[0.05, 11.69]	0.048	0.40
Sleep Hours	7.42 ± 1.1	7.52 ± 0.9	7.33 ± 1.2	[−0.45, 0.83]	0.548	0.18

Note. M = mean; SD = standard deviation; 95% CI = 95% confidence interval of the mean difference; *d* = effect size (Cohen’s *d*). *p*-values correspond to independent-samples *t*-tests.

**Table 4:** Pearson Correlation Matrix (r) with 95% Confidence Intervals.

Variables	1	2	3	4	5
1. Mental Health (MHI-5)	—				
2. Status (PPP = 1)	0.321 * [0.03, 0.56]	—			
3. Sleep Hours	0.435 ** [0.16, 0.65]	0.112 [−0.19, 0.39]	—		
4. Motivation	0.451 ** [0.18, 0.66]	0.204 [−0.10, 0.47]	0.312 * [0.02, 0.56]	—	
5. Training Duration (Isolation)	0.382 ** [0.10, 0.61]	0.285 * [0.01, 0.53]	0.214 [−0.09, 0.48]	0.488 ** [0.22, 0.68]	—

Note. r = Pearson correlation coefficient; 95% CI = 95% confidence interval. * *p* < 0.05; ** *p* < 0.01.

**Table 5:** Multiple Linear Regression Model for Mental Health.

Predictor Variables	*B*	β	CI 95% for *B*	t	*p*	VIF
(Constant)	24.18	—	[10.21, 38.15]	3.52	0.001	—
Sleep Hours	4.32	0.351	[1.18, 7.46]	2.78	0.008	1.12
Status (PPP = 1)	5.84	0.315	[0.80, 10.88]	2.34	0.024	1.21
Motivation	2.18	0.278	[0.09, 4.27]	2.12	0.041	1.30
Training Duration (Isolation)	2.95	0.241	[−0.01, 5.91]	1.99	0.051	1.25

Note. *B* = unstandardized coefficient; β = standardized coefficient; 95% CI = 95% confidence interval; VIF = Variance Inflation Factor; R^2^ = coefficient of determination of the model. *p* < 0.05.

## Data Availability

The raw data supporting the conclusions of this article will be made available by the authors on request.
